# Long non-coding RNA HOTAIR, a c-Myc activated driver of malignancy, negatively regulates miRNA-130a in gallbladder cancer

**DOI:** 10.1186/1476-4598-13-156

**Published:** 2014-06-23

**Authors:** Ming-zhe Ma, Chun-xiao Li, Yan Zhang, Ming-zhe Weng, Ming-di Zhang, Yi-yu Qin, Wei Gong, Zhi-wei Quan

**Affiliations:** 1 Department of General Surgery, Xinhua Hospital, Shanghai Jiaotong University School of Medicine, 1665 Kongjiang Road, Shanghai 200092, People’s Republic of China; 2 Department of General Surgery, Taixing people’s Hospital, Yangzhou University School of Medicine, Jiangsu Province, China; 3 Department of Dermatology, Xinhua Hospital, Shanghai Jiaotong University School of Medicine, Shanghai, China; 4 Department of Gastroenterology, Yijishan Hospital affiliated to Wannan medical College, Wuhu, Anhui, China

**Keywords:** Long non-coding RNA, HOTAIR, miRNA-130a, c-Myc, Gallbladder cancer

## Abstract

**Background:**

Protein coding genes account for only about 2% of the human genome, whereas the vast majority of transcripts are non-coding RNAs including long non-coding RNAs. A growing volume of literature has proposed that lncRNAs are important players in cancer. HOTAIR was previously shown to be an oncogene and negative prognostic factor in a variety of cancers. However, the factors that contribute to its upregulation and the interaction between HOTAIR and miRNAs are largely unknown.

**Methods:**

A computational screen of HOTAIR promoter was conducted to search for transcription-factor-binding sites. HOTAIR promoter activities were examined by luciferase reporter assay. The function of the c-Myc binding site in the HOTAIR promoter region was tested by a promoter assay with nucleotide substitutions in the putative E-box. The association of c-Myc with the HOTAIR promoter *in vivo* was confirmed by chromatin immunoprecipitation assay and Electrophoretic mobility shift assay. A search for miRNAs with complementary base paring with HOTAIR was performed utilizing online software program. Gain and loss of function approaches were employed to investigate the expression changes of HOTAIR or miRNA-130a. The expression levels of HOTAIR, c-Myc and miRNA-130a were examined in 65 matched pairs of gallbladder cancer tissues. The effects of HOTAIR and miRNA-130a on gallbladder cancer cell invasion and proliferation was tested using *in vitro* cell invasion and flow cytometric assays.

**Results:**

We demonstrate that HOTAIR is a direct target of c-Myc through interaction with putative c-Myc target response element (RE) in the upstream region of HOTAIR in gallbladder cancer cells. A positive correlation between c-Myc and HOTAIR mRNA levels was observed in gallbladder cancer tissues. We predicted that HOTAIR harbors a miRNA-130a binding site. Our data showed that this binding site is vital for the regulation of miRNA-130a by HOTAIR. Moreover, a negative correlation between HOTAIR and miRNA-130a was observed in gallbladder cancer tissues. Finally, we demonstrate that the oncogenic activity of HOTAIR is in part through its negative regulation of miRNA-130a.

**Conclusion:**

Together, these results suggest that HOTAIR is a c-Myc-activated driver of malignancy, which acts in part through repression of miRNA-130a.

## Introduction

Gallbladder cancer (GBC) is the common biliary tract cancer and the fifth most common gastrointestinal malignancy [1]. The outcome of patients with more advanced disease is dismal with 5-year survival rates ranging from 20% to 40% [2]. Chemo-resistance and progression are the most remarkable characteristics of GBC [3]. Although previous studies identified accumulated genomic damage promote the progression of GBC [4-6], the pathophysiological mechanism contributing to GBC are still largely unknown. Therefore, it is of paramount importance to understand the roles of novel molecules involved in this process.

It is well known that protein-coding genes account for only about 2% of the human genome, whereas the vast majority of transcripts are non-coding RNAs [7], and both microRNAs (21–24 nt) (miRNAs) and long ncRNAs (>200 nt) (lncRNAs) are now emerging as mammalian transcription key regulators [8,9]. A number of studies over the past five years have identified a large number of miRNAs differentially expressed in GBC that are correlated with malignancy [10-12]. A growing volume of literature has proposed that lncRNAs are important players in cancer [13-15]. Although it is well known that miRNAs can target a number of protein-coding genes, little is known whether miRNAs/lncRNAs can also target lncRNAs/miRNAs. Recently, a competitive RNA (ceRNA) hypothesis has been proposed and several studies have suggested the interaction between lncRNA and miRNA in cancer [16-19], adding another piece of puzzle to the miRNA and lncRNA regulatory networks.

HOTAIR, is a 2158-bp lnRNA that is located at the antisense strand of the HOXC gene locus in chromo-some 12, flanked by HOXC11 and HOXC12. HOTAIR is negative prognostic factor for a variety of carcinomas, and HOTAIR expression levels are correlated with tumor metastases while loss of HOTAIR has been linked with decrease in cancer invasiveness [15,20,21]. The activity of HOTAIR is due, in part, to its induction of genome-wide targeting of the polycomb repressive complex 2 (PRC2), leading to an altered methylation of histone H3 lysine 27 (H3K27) and genes expression. In this study, we also find that HOTAIR is upregulated in GBC compared to adjacent normal tissues. However, although HOTAIR has been shown to play a vital role in cancer, the factors that contribute to its upregulation and the interaction between HOTAIR and miRNAs are largely unknown.

In the present study, we demonstrate that HOTAIR is a direct target of c-Myc through interaction with putative c-Myc target response element (RE) in the upstream region of HOTAIR. Our data also reveal that HOTAIR negatively regulates miRNA-130a and the oncogenic activity of HOTAIR is, at least in part, through the negative regulation of miRNA-130a, which may function as a part of the “competitive endogenous RNAs (ceRNA)” network [22].

## Material and method

### Cell culture

Four human GBC cell lines (GBC-SD, SGC-996,NOZ and EH-GB2) were used in this study. GBC-SD and SGC-996 were purchased from Cell Bank of the Chinese Academy of Science (Shanghai, China). NOZ was purchased from the Health Science Research Resources Bank (Osaka, Japan). EH-GB2 was a generous gift from Eastern Hepatobiliary Surgical Hospital and Institute, The Second Military University, Shanghai [23]. The cell lines were cultured in Dulbecco’s modified Eagle’s medium (DMEM, Gibco BRL), containg 10% fetal calf serum (FBS, HyClone) as well as 100 U/ml penicillin and 100 μg/ml streptomycin (Invitrogen). Cells were maintained in a humidified incubator at 37°C in the presence of 5% CO_2_. All cell lines have been passaged for fewer than 6 months.

### Patients and samples

Sixty-five GBC tissue samples and neighboring noncancerous gallbladder tissue samples (collected postoperatively from August 2007 to September 2010) used in this study were obtained from Xinhua Hospital, Shanghai, China. Upon removal of the surgical specimen, surgical pathology faculty performed a gross analysis of the specimen and selected gallbladder tissues that appeared to be cancerous and gallbladder tissues that appeared to be normal for analysis. Each sample was snap-frozen in liquid nitrogen and stored at -80°C prior to RNA isolation and qRT-PCR analysis. All patients recruited to this study did not receive any pre-operative treatments. GBC patients were staged according to the tumor node metastasis (TNM) staging system (the 7th edition) of the American Joint Committee on Cancer (AJCC) staging system. The data do not contain any information that could identify the patients. All patients provided written informed consent. Complete clinicopathological follow-up data of the GBC patients from which the specimens were collected were available. This study was approved by the Human Ethics Committee of Xinhua Hospital at Shanghai Jiaotong University (Shanghai, China).

### RNA preparation, reverse transcription and qPCR

Total RNA from tissues and cells was extracted using Trizol reagent (Invitrogen, CA). RNA was reversed transcribed into cDNAs using the Primer-Script™ one step RT-PCR kit (TaKaRa, Dalian, China). The cDNA template was amplified by real-time RT-PCR using the SYBR® Premix Dimmer Eraser kit (TaKaRa, Dalian, China). GAPDH was used as an internal control, and mRNA values were normalized to GAPDH. Real-time RT-PCR reactions were performed by the ABI7500 system (Applied Biosystems, CA). The relative expression fold change of mRNAs was calculated by the 2^-ΔΔCt^ method. The primer sequences were listed in the Additional file 1.

### Plasmid construction

Expression plasmids for HOTAIR, c-Myc or corresponding mutants of HOTAIR by mutating the has-miRNA-130a seed region binding site (seed sequence binding fragment 5’-GACTTTGCACT -3’ changed to 5’-TTGTAACGTGA-3’) were created using PCR amplification with human genomic DNA as templates. The primer sequences used are listed in Additional file 1. The PCR product was verified and subcloned into the mammalian expression vector pcDNA3.1 (Invitrogen). Plasmids were transfected into cells with lipofectamine 2000 (Invitrogen, CA).

The promoter region of HOTAIR was PCR-amplified by PrimerStar polymerase (TaKaRa) with the primers 5’-ACTGGTACCTAAGCGGAGAGAGTCCC-3’ (forward) and 5’-ACTAAGCTTGAGTCAGAGTTCCCCAC-3’ (reverse) and was subcloned into the pGL3 basic firefly luciferase reporter. The pGL3 construct containing the HOTAIR promoter with a point mutation in the E-box element was PCR-amplified by PrimerStar polymerase (TaKaRa) with the primers 5’-ACTGGTACCTAAGCGGAGAGAGTCCCACACAGG-3’ (forward) and 5’-ACTAAGCTTGAGTCAGAGTTCCCCAC-3’ (reverse) and was subcloned into the pGL3 basic firefly luciferase reporter.

### Cell transfection

HOTAIR siRNA, c-Myc siRNA and Allstars Negative Control siRNA were purchased from Qiagen, Hilden, Germany. Target sequences are listed in the Additional file 1. Hsa-miRNA-130a mimic/negative control mimic and hsa-miRNA-130a inhibitor/negative control inhibitor were purchased from Genechem, shanghai, China.

Cells were grown on six-well plates to confluency and transfected using Lipofectamine 2000 (Invitrogen) according to the manufacturer’s instructions. Forty-eight hours after transfection, cells were harvested for qRT-PCR or western blot analyses.

### Luciferase reporter assay

For the promoter activity of HOTAIR, the promoter/luciferase reporter construct and pcDNA3.1-c-Myc or c-Myc siRNA were cotransfected into cultured cells by Lipofectamine-mediated gene transfer. Each sample was cotransfected with the pRL-TK plasmid, which expressed renilla luciferase to monitor transfection efficiency (Promega, Madison, WI, USA). The relative luciferase activity was normalized with renilla luciferase activity.

For the dual luciferase activity, LncRNA-HOTAIR (lncRNA-HOTAIR-wt) or its mutant devoid of specific miRNA binding sites (lncRNA-HOTAIR-mu) was cloned into 3’UTR of the Renilla luciferase gene in the vector pRL-TK (Promega, Madison, WI, USA). Each plasmid was transfected into cells, together with specific miRNAs mimics or with a negative control mimic (RiboBio, Guangzhou, China). Firefly luciferase gene in the vector pGL3-control (Promega) was used as a control for transfection efficiency. Luciferase assays were performed using the dual-luciferase reporter assay system kit (Promega) according to the manufacturer’s instructions. Luciferase expression was analyzed by Modulus single-tube multimode reader (Promega). The relative luciferase expression equals the expression of Renilla luciferase (pRL-TK) divided by the expression of firefly luciferase.

### Chromatin immunoprecipitation (ChIP) assay

Chromatin immunoprecipitation (ChIP) assays were performed according to the EZ ChIP Chromatin Immunoprecipitation Kit (Millipore, Bedford, MA, USA). Briefly, crosslinked chromatin was sonicated into 200- to 1,000-bp fragments. Anti-c-Myc and anti-Max antibodies (Cell Signal Technology, USA) were used to precipitate DNA–protein complexes. Normal mouse immunoglobulin G (IgG) was used as a negative control. ChIP-derived DNA was quantified using qRT-PCR with SYBR-Green incorporation (Applied Biosystems, Foster City, CA, USA). The primers are listed in Additional file 1.

### Electrophoretic mobility shift assay (EMSA)

The following double-stranded oligonucleotides were used (wild type and mutant binding sites are underlined): E-box, 5’-CGAGCGCAGTGGCGCATGGCTGTAATCCCA-3’; E-box mutant, 5’-CGAGCGCAGTGGCATGGGGCTGTAATCCCA-3’. Oligonucleotide labeling was performed using the Biotin 3’ End Labeling Kit (Pierce, USA). EMSA was performed using a light shift chemiluminescent EMSA kit (Pierce). Nuclear proteins from gallbladder cancer cells were prepared using NE-PER nuclear and cytoplasmic extraction reagents (Pierce). Nuclear extract proteins (4 μg) were incubated in with binding buffer (2.5% glycerol, 5 mM MgCl_2_, 0.05% NP-40, 1 μg poly(dI-dC), 10 mM Tris, 50 mM KCl, 1 mM DTT, pH 7.5). Samples were electrophoresed on a 6% polyacrylamide gel in 0.5× TBE buffer (45 mM Tris, 45 mM boric acid, 1 mM EDTA, pH 8.3). For competition assays, samples were preincubated with a 200-fold excess (4 pmol) of the unlabeled wild type competitors for 20 min. For the supershift reaction, 1 μg of each anti-c-Myc antibody was preincubated with the nuclear extracts in the absence of poly (dI · dC) for 1 hour at 4°C. Subsequently, poly (dI · dC) was added and incubated for 5 min, followed by the incubation of the biotin labeled probes (20 fmol) for 20 min. Samples were separated by electrophoresis on a 6% non-denaturing acrylamide gel in 0.5 × TBE, transferred to positively charged nylon membranes, and visualized by streptavidin-horseradish peroxidase followed by chemiluminescent detection.

### Northern blot analysis

Total RNA (15 μg) from samples were separated on 15% denaturing polyacrylamide gels, transferred onto GeneScreen Plus membranes (PerkinElmer), and hybridized using UltraHyb-Oligo buffer (Ambion). Oligonucleotides complementary to mature miR-130a (5’-AGCAAAAATGTGCTAGTGCCAAA-3’) were end-labeled with T4 Kinase (Invitrogen) and used as probes. Following hybridization at 42°C overnight, the membranes washed twice in 0.1 × SSPE and 0.1% SDS at 42°C for 15 min each. Membranes were then exposed to a storage phosphor screen (GE Healthcare Bio-Sciences) for 8 h and imaged using a Typhoon 9410 Variable Mode Imager (GE Healthcare Bio-Sciences). Northern blots hybridized with a 5S ribosomal RNA (rRNA) cDNA were used as controls.

### RNA pull-down assay

To determine whether HOTAIR is associated with the RNA-induced silencing complex (RISC) complex, we performed RNA pull-down assay using synthesized biotin-labeled HOTAIR as a probe and then detected Ago 2 from the pellet by western or detected miRNA-130a by quantitative RT-PCR (qRT-PCR).

RNA pull-down were performed as described previously [16]. Briefly, the DNA fragment covering has-miRNA-130a seed region binding site of HOTAIR was PCR-amplified using a T7 containing primer and then cloned into pCR8 (Invitrogen). In addition, lncRNA loc285194 [16] was also cloned and used in RNA Pull-Down Assay as a positive control. The resultant plasmid DNA was linearized with restriction enzyme Not I. Biotin-labeled RNAs were *in vitro* transcribed with the Biotin RNA Labeling Mix (Roche Diagnostics, Indianapolis, IN) and T7 RNA polymerase (Roche), treated with RNase-free DNase I (Roche), and purified with the RNeasy Mini Kit (Qiagen, Inc.,Valencia, CA). Cell nuclear extract (2 ug) was mixed with biotinylated RNA (100 pmol). Washed Streptavidin agarose beads (100 ml) were added to each binding reaction and further incubated at room temperature for 1 h. Beads were washed briefly three times and boiled in SDS buffer, and the retrieved protein was detected by standard western blot technique.

The Ago2 antibodies used for RIP are purchased from Abcam (Abcam, Cambridge, MA). The coprecipitated RNAs were detected by reverse transcription PCR. Total RNAs and controls were also assayed to demonstrate that the detected signals were from RNAs specifically binding to Ago2.

### Western blot

Western blot analysis to assess c-Myc, Ago2 and GADPH expression was carried out as described previously [13]. GADPH primary antibodies were purchased from Sigma (MO, USA).

### Cell invasion assay

For the invasion assays, 48 h after transfection, 5 × 10^4^ cells in serum-free media were placed into the upper chamber of an insert (8.0 μm, Millipore, MA) coated with Matrigel (Sigma, USA). The chambers were then incubated for 24 h in culture medium with 10% FBS in the bottom chambers before examination. The cells on the upper surface were scraped and washed away, whereas the invaded cells on the lower surface were fixed and stained with 0.05% crystal violet for 2 h. Finally, invaded cells were counted under a microscope and the relative number was calculated. Experiments were independently repeated in triplicate.

### Flow cytometric analysis

Cells were seeded at a density of 1 × 10^6^ cells/well in six-well plates. After 24 h, cells were washed with PBS and fixed in ice-cold 70% ethanol for 1 h and then treated with 100 uL of 50 mg/L propidium iodide for 30 min at 4°C in the dark. The cell-cycle profiles were assayed using the Elite ESP flow cytometer at 488 nm, and data were analyzed with the CELL Quest software (BD Biosciences,San Jose, CA, USA).

### Statistical analysis

All statistical analyses were performed using SPSS 17.0 (SPSS, Chicago, USA). The gene expression level of HOTAIR in tumors was compared with adjacent normal tissues utilizing paired samples *t*-test. The expression differences between high/low grades, high/low stages, cell lines, the expression changes after transfection, luciferase activity, cell cycle and cell migration assays were analyzed using independent samples *t*-test. All data were presented as mean ± standard error. A two-sided *p* value of less than 0.05 was considered to be statistically significant.

## Results

### HOTAIR is upregulated in gallbladder cancer tissues

**Figure 1:**
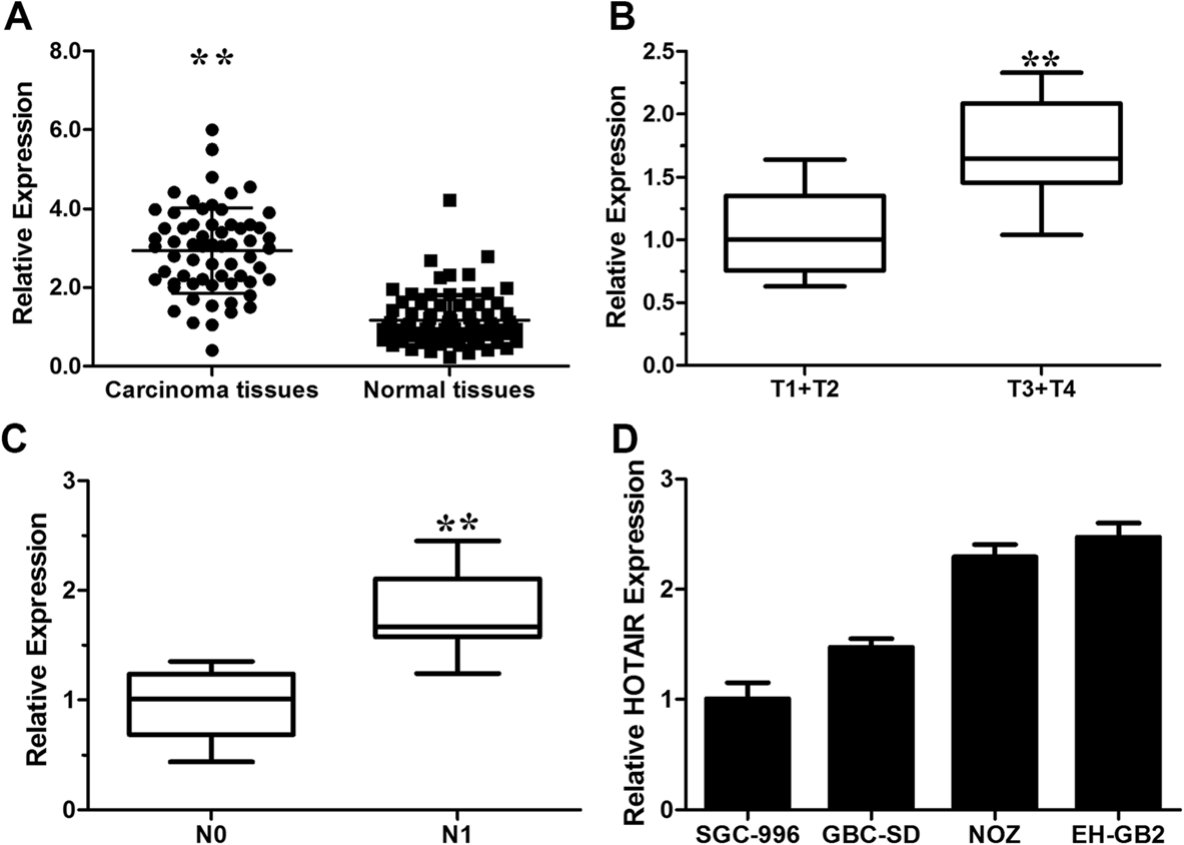
**HOTAIR expression in gallbladder cancer and its clinical significance. (A)** Difference in expression levels of HOTAIR expression levels between gallbladder cancer tissues and matched non-tumor gallbladder tissues. The expression of HOTAIR was normalized to GADPH. The statistical differences between samples were analyzed with paired samples *t*-test (n = 65, *p* < 0.0001). **(B)** Relationship between HOATIR expression and primary tumor growth (*p* < 0.0001). **(C)** Relationship between HOATIR expression and lymph node metastasis (*p* < 0.0001). **(D)** Expression level of HOTAIR in four gallbladder cancer cell lines. The statistical differences between groups were analyzed using independent samples *t*-test. *p < 0.05; **p < 0.01.

The expression level of HOTAIR was examined using real-time PCR in 65 pairs of gallbladder cancer tissues and matched adjacent normal tissues. Detailed clinical features are presented in Table 1. As illustrated in Figure 1A, the HOTAIR transcripts were expressed at higher levels in the tumor tissues compared with adjacent normal tissues (*p* < 0.0001, Figure 1A), indicating that HOTAIR was frequently up-regulated in GBC. Next, we examined the expression level of HOTAIR with clinical characteristics in 65 gallbladder cancer tissues (Figure 1B, C). HOTAIR was more highly expressed in tumors extending beyond the gallbladder (T3 + T4) compared with tumors only detected in the gallbladder (T1 + T2) (Figure 1B) and more highly expressed in tumors spread to regional lymph nodes (N1) compared with tumors localized only in the gallbladder (N0) (Figure 1C). We then examined the expression level of HOTAIR in a series of gallbladder cancer cell lines (Figure 1D). We selected gallbladder cancer cell line GBC-SD as our experimental model as GBC-SD harbors a moderate expression level of HOTAIR, which makes it easier for manipulation.

**Table 1:** Clinicopathological profiles of 65 primary gallbladder cancer patients

**Sex(male/female)**	**40 (62%)/25 (38%)**
**Median age**	**64 years (ranage, 40 to 78 years)**
*T category*	
T1	3 (5%)
T2	9 (14%)
T3	46 (71%)
T4	7 (10%)
*N category*	
N0	36 (55%)
N1	29 (45%)
*M category*	
M0	65 (100%)
M1	0 (0%)
*Histology*	
Well differentiated	16 (24%)
Moderate differentiated	42 (65%)
Poorly differentiated	7 (11%)
*Lymphatic permeation*	
N0	37 (57%)
N1	28 (43%)

### c-Myc directly binds to the promoter regions of HOTAIR and upregulates its expression

To determine how transcription of HOTAIR was regulated, we then performed a search for possible transcription factor-binding sites using online software programs.

**Figure 2:**
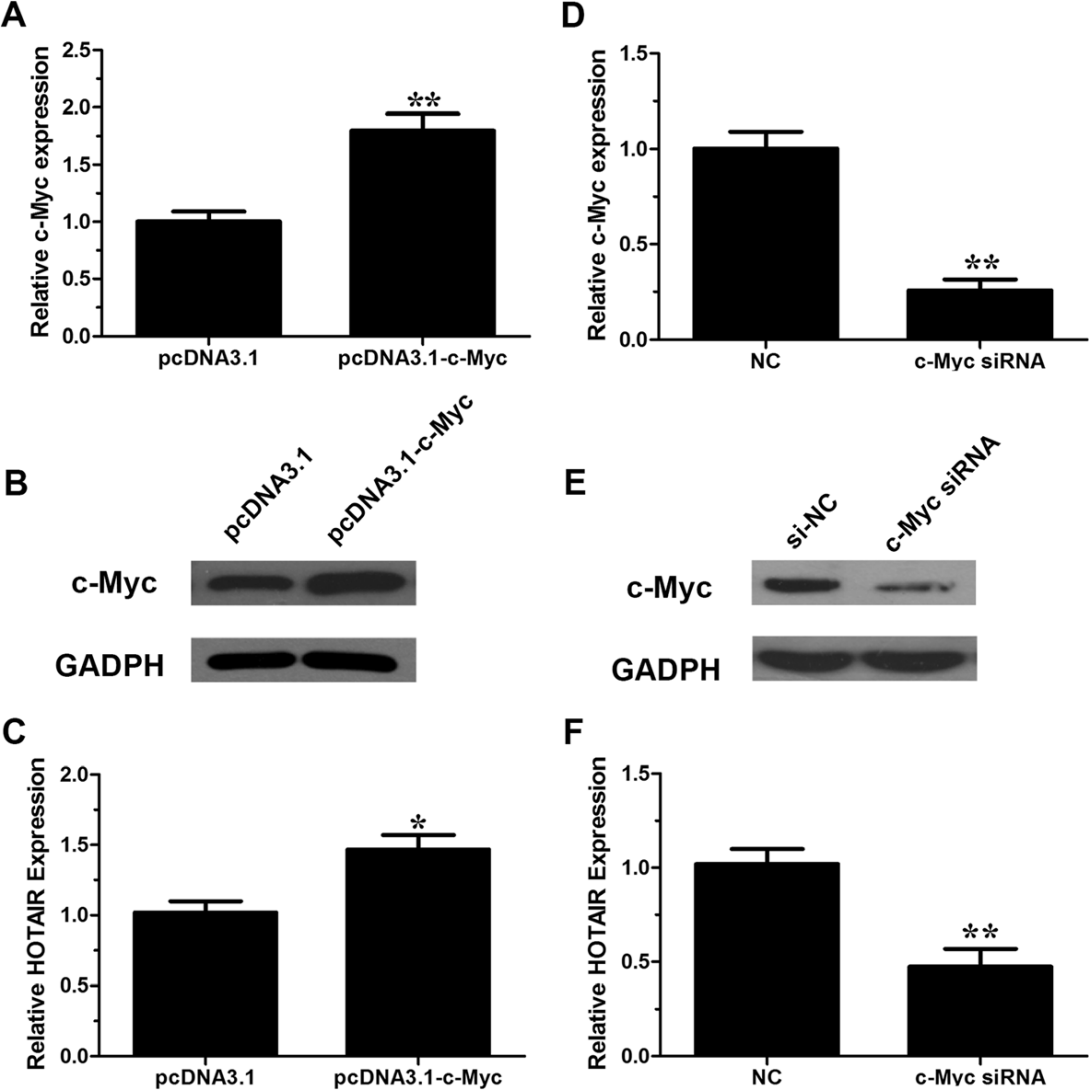
**The expression changes of c-Myc or HOTAIR after transfection of c-Myc siRNA or pcDNA3.1-c-Myc in GBC-SD cells.** The relative mRNA expression levels were evaluated with real-time qPCR. c-Myc protein levels were determined using Western blot assay. Each experiment was performed in triplicate. **(A)** pcDNA3.1-c-Myc markedly upregulated the expression of c-Myc at mRNA levels. **(B)** Representative images of western blot results indicated pcDNA3.1-c-Myc significantly upregulated the expression of c-Myc at protein levels. **(C)** pcDNA3.1-c-Myc significantly upregulated the expression of HOTAIR at mRNA levels. **(D)** c-Myc siRNA significantly downregulated the expression of c-Myc at mRNA levels. **(E)**) Representative images of western blot results indicated c-Myc siRNA significantly downregulated the expression of c-Myc at protein level. **(F)** c-Myc siRNA significantly down-regulated the expression of HOTAIR at mRNA levels. Error bars represent S.E.M., n = 3. *p < 0.05; **p < 0.01.

**Figure 3:**
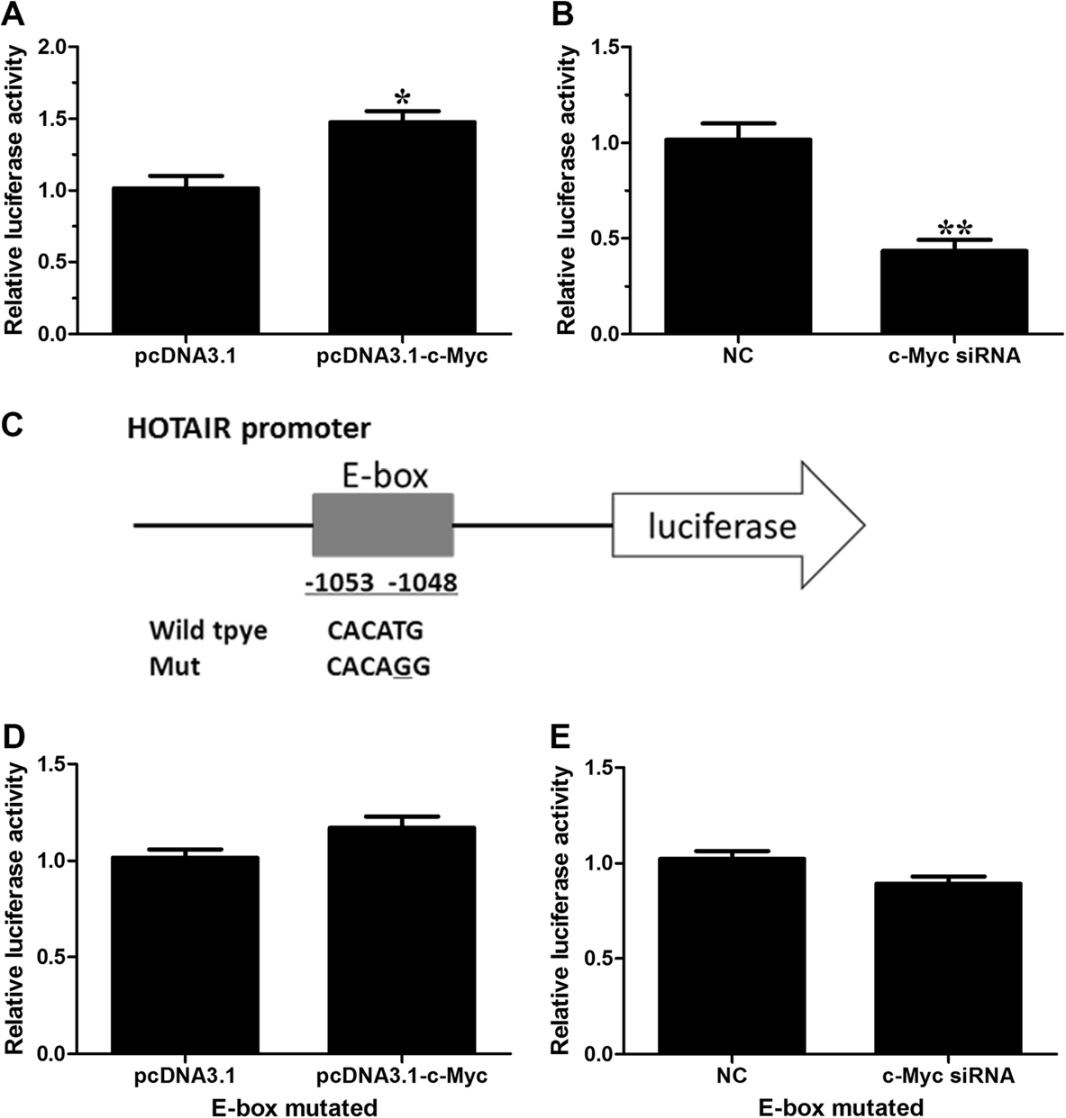
**c-Myc regulates HOTAIR promoter activity, depending on E-box element. (A)** Dual luciferase assay on GBC-SD cells cotransfected with firefly luciferase constructs containing the HOTAIR promoter and pcDNA3.1 or pcDNA3.1-c-Myc. **(B)** Dual luciferase assay on GBC-SD cells cotransfected with firefly luciferase constructs containing the HOTAIR promoter and c-Myc siRNA or the control siRNA. **(C)** schematic of the HOTAIR-promoter-luciferase construct is depicted with locations of the E-box element and sequences of point mutation. **(D, E)** Dual luciferase assay on GBC-SD cells cotransfected with firefly luciferase constructs (mutant at E-box element) and pcDNA3.1-c-Myc **(D)** or c-Myc siRNA **(E)**. All of the transfection was performed in triplicates. The values are presented as the mean ± S.E.M. of the ratio of firefly luciferase activity to renilla luciferase activity and are representative of at least three independent experiments. Data are shown as the mean ± S.E.M, based on at least three independent experiments. *p < 0.05; **p < 0.01.

**Figure 4:**
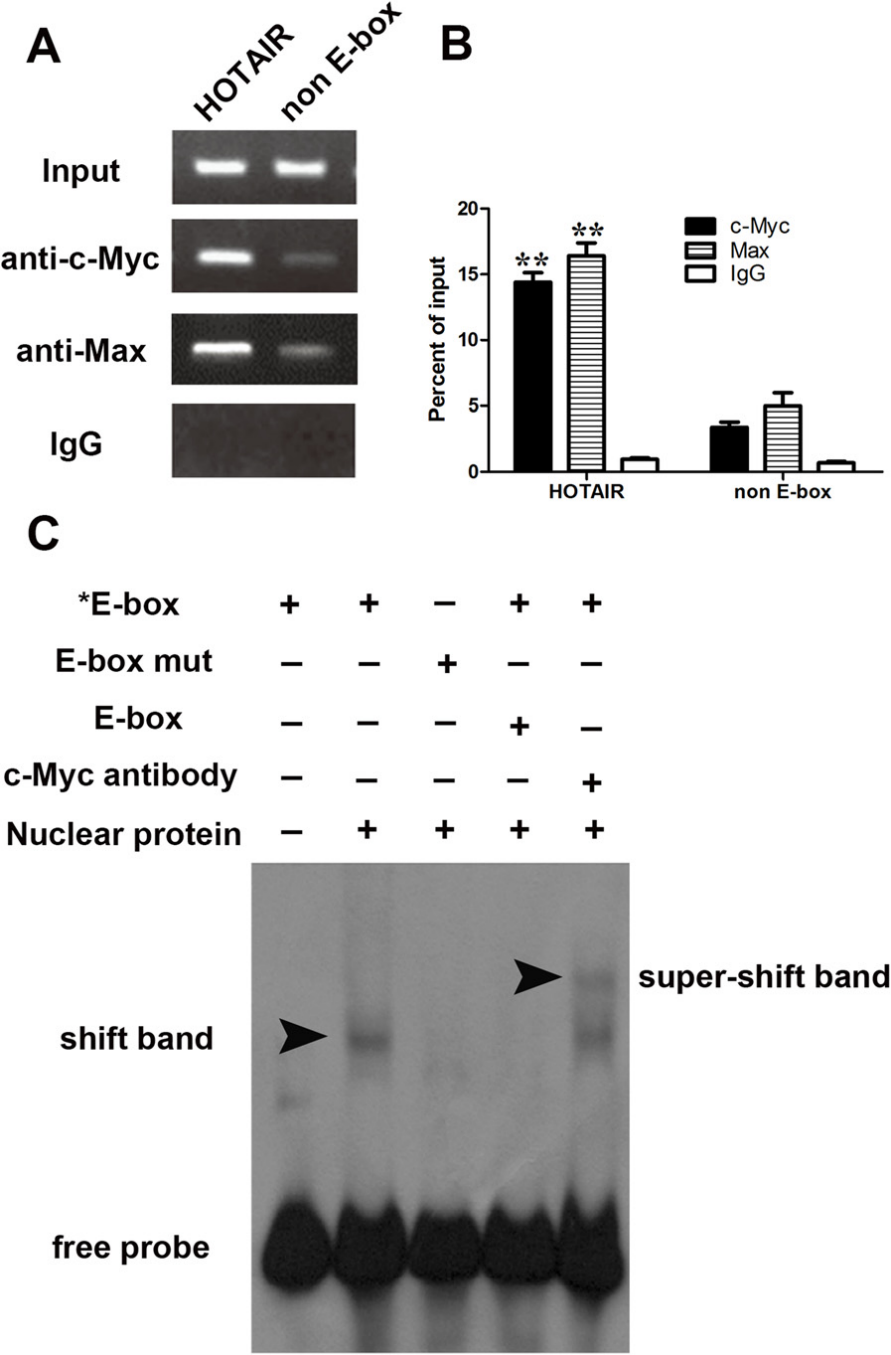
**Confirmation of the binding of c-Myc and Max at the promoter region of HOTAIR. (A)** c-Myc binding at the promoter region of HOTAIR containing the E-box element and a random region in HOTAIR promoter region (does not contain an E-box, negative control) was assessed by chromatin immunoprecipitation (ChIP). **(B)** ChIP-derived DNA was amplified by qRT-PCR using specific primers. The levels of qPCR products are expressed as a percentage of input DNA. **(C)** EMSA showed the interaction of c-Myc with the lncRNA-HOTAIR promoter in vitro. The symbol “*” means the oligonucleotides labled by biotin. Data are shown as the mean ± S.E.M, based on at least three independent experiments. *p < 0.05; **p < 0.01.

MatInspector (http://www.genomatix.de/online_help/help_matinspector/matinspector_help.html) and TFSEARCH (http://www.cbrc.jp/research/db/TFSEARCH.html) in the promoter region of HOTAIR and found that one putative E-box element at ~1053 upstream of HOTAIR which could be recognized by c-Myc (Additional file 2: Figure S1). c-Myc has been reported to be an oncoprotein and is deregulated in gallbladder cancer [5,24].Firstly, we would like to evaluate whether c-Myc regulates the expression of HOTAIR. We manipulated the expression of c-Myc by transfecting pcDNA3.1-c-Myc (Figure 2A, B) or siRNA into gallbladder cancer cells GBC-SD (Figure 2D, E). At 48 h after treatment, we measured the expression level of HOTAIR. Our results indicated that ectopic expression of c-Myc enhanced the expression of HOTAIR (Figure 2C) while inhibition of c-Myc decreased the expression of HOTAIR (Figure 2F).To further explore the mechanism of c-Myc-induced HOTAIR upregulation in GBC cells, we cloned the promoter of HOTAIR into the pGL3 basic firefly luciferase reporter (Promega) and cotransfected the construct with pcDNA3.1-c-Myc or c-Myc specific siRNA into GBC-SD cells. Overexpression of c-Myc dramatically increased the luciferase activity of construct while inhibition of c-Myc decreased the luciferase activity (Figure 3A, B). We then mutated the E-box element, as depicted in Figure 3C, and cotransfected cells with pcDNA3.1-c-Myc or c-Myc siRNA. Our results indicated that mutation of the E-box element abolished the effects of c-Myc on the promoter activity of HOTAIR (Figure 3D, E). The data suggest that c-Myc may activate the HOTAIR promoter. c-Myc binds to DNA sequence elements called E-boxes to allow for target gene transcription with its obligate heterodimerization partner Max. In a ChIP assay, c-Myc and Max immunoprecipitates were highly enriched in the DNA fragments compared with negative control IgG immunoprecipitates (Figure 4A). A random region which does not contain an E-box site, showed no significant enrichment (Figure 4B). To confirm the physical interaction of c-Myc with the putative E-box element in HOTAIR promoter, we performed the electrophoretic mobility shift assay using biotin-labeled, synthetic double-stranded oligonucleotides corresponding to the E-box. As shown in Figure 4C, a binding complex was formed between c-Myc and the labeled wild-type HOTAIR oligonucleotides. However, the binding was not observed with mutated probe. Furthermore, the bands were supershifted by specific c-Myc antibodies. Together, these results suggest that c-Myc interacts with the c-Myc responsive element in the HOTAIR promoter to induce its transcription.

### Reciprocal repression of miRNA-130a and HOTAIR

**Figure 5:**
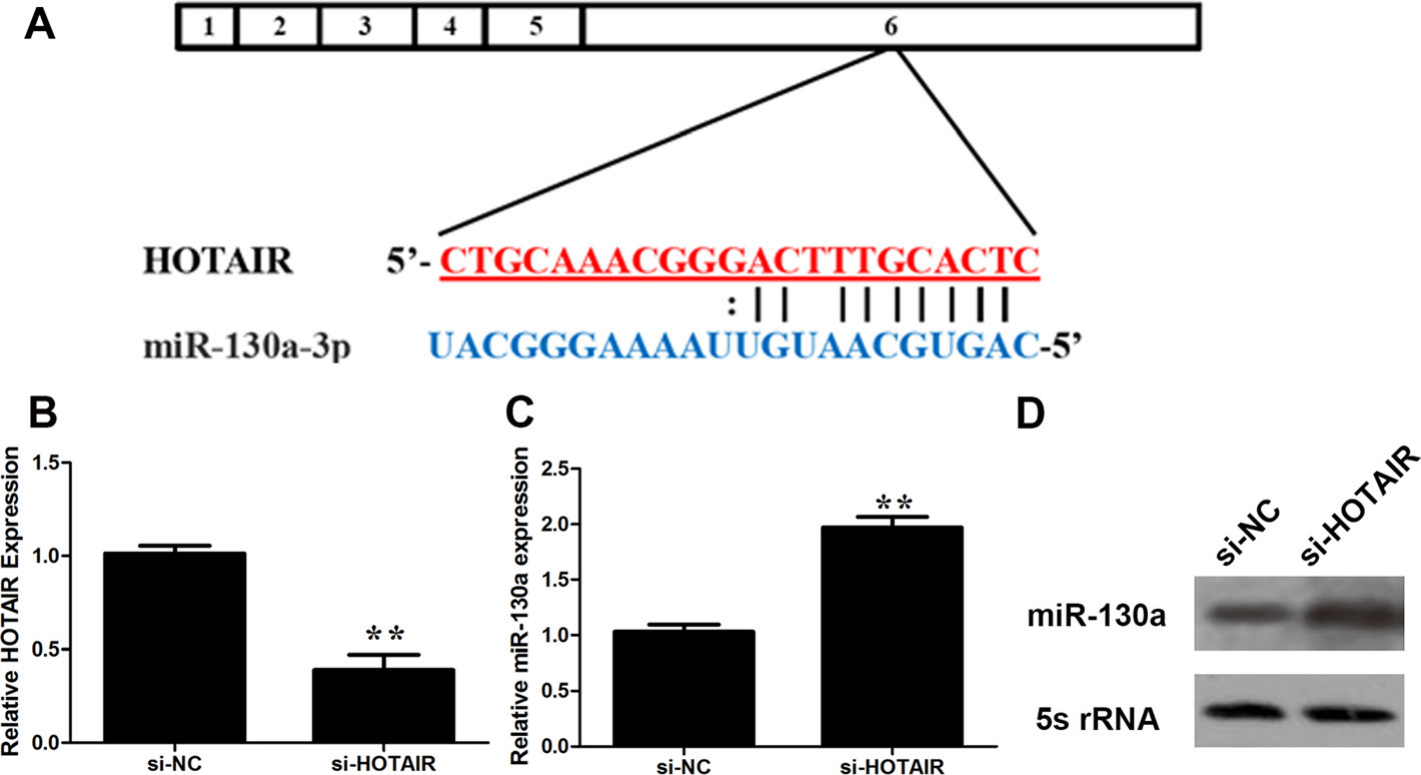
**Identification of miR-130a as a target of HOTAIR. (A)** Alignment of potential HOTAIR base pairing with miR-130a as identified by Starbase v2.0 (http://starbase.sysu.edu.cn/mirLncRNA.php). HOATIR (top) consist of 6 exons**,** where the putative binding site is in exon 6. **(B)** HOTAIR specific siRNA reduced the endogenous HOTAIR mRNA level. **(C, D)** Upregulation of miR-130a by si-HOTAIR detected by RT-PCR **(C)** and northern blot **(D)**. GBC-SD cells were transfected with control siRNA or si-HOTAIR, and total RNA was isolated 48 h after transfection. Error bars represent S.E.M., n = 3. *p < 0.05; **p < 0.01.

**Figure 6:**
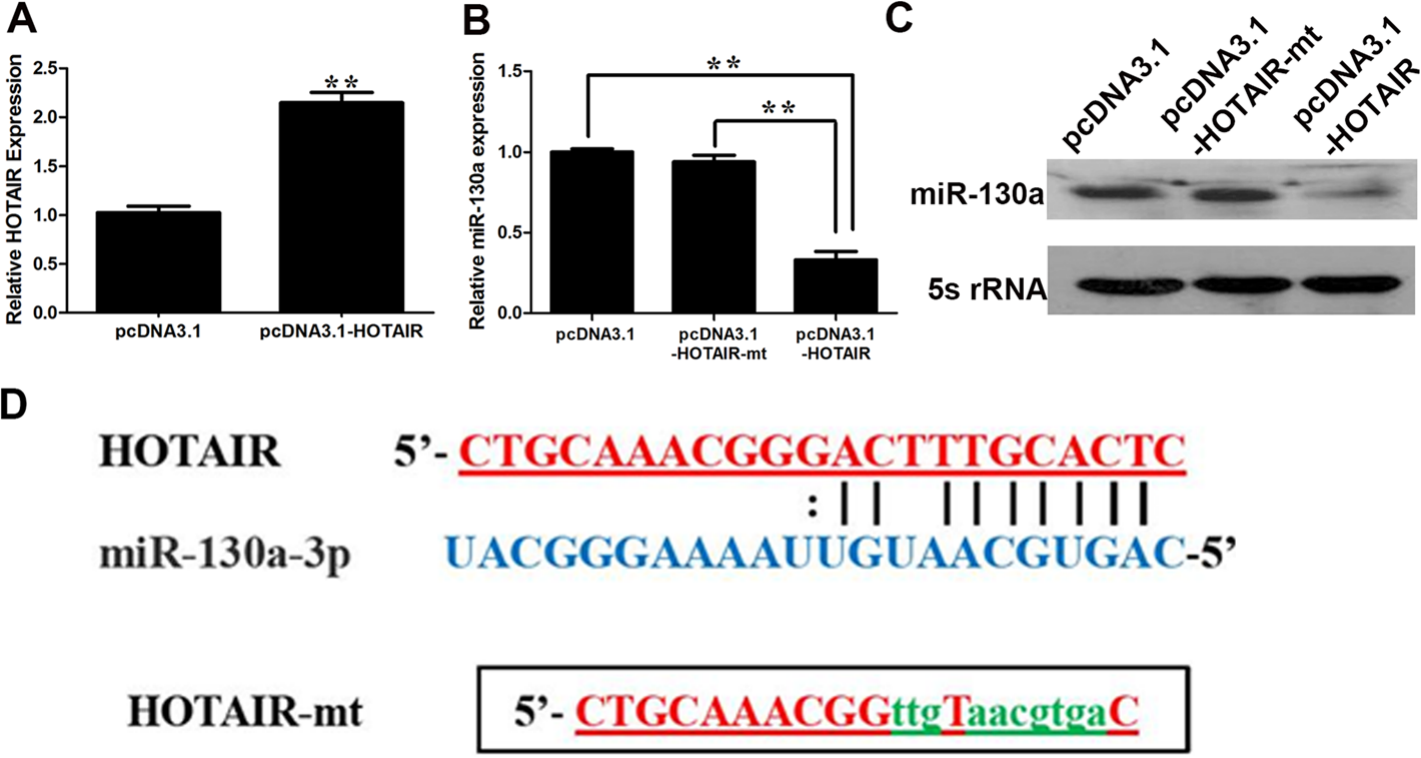
**Identification of miR-130a as a target of HOTAIR. (A)** pcDNA3.1-HOTAIR upregulated the HOTAIR mRNA level. **(B, C)** Downregulation of miR-130a by ectopic expression of HOTAIR detected by RT-PCR **(B)** and northern blot **(C)**. GBC-SD cells were transfected with vector control or HOTAIR or mutant HOTAIR, and total RNA was isolated 48 h after transfection. **(D)** The mutant HOTAIR at putative binding site. Error bars represent S.E.M., n = 3. *p < 0.05; **p < 0.01.

**Figure 7:**
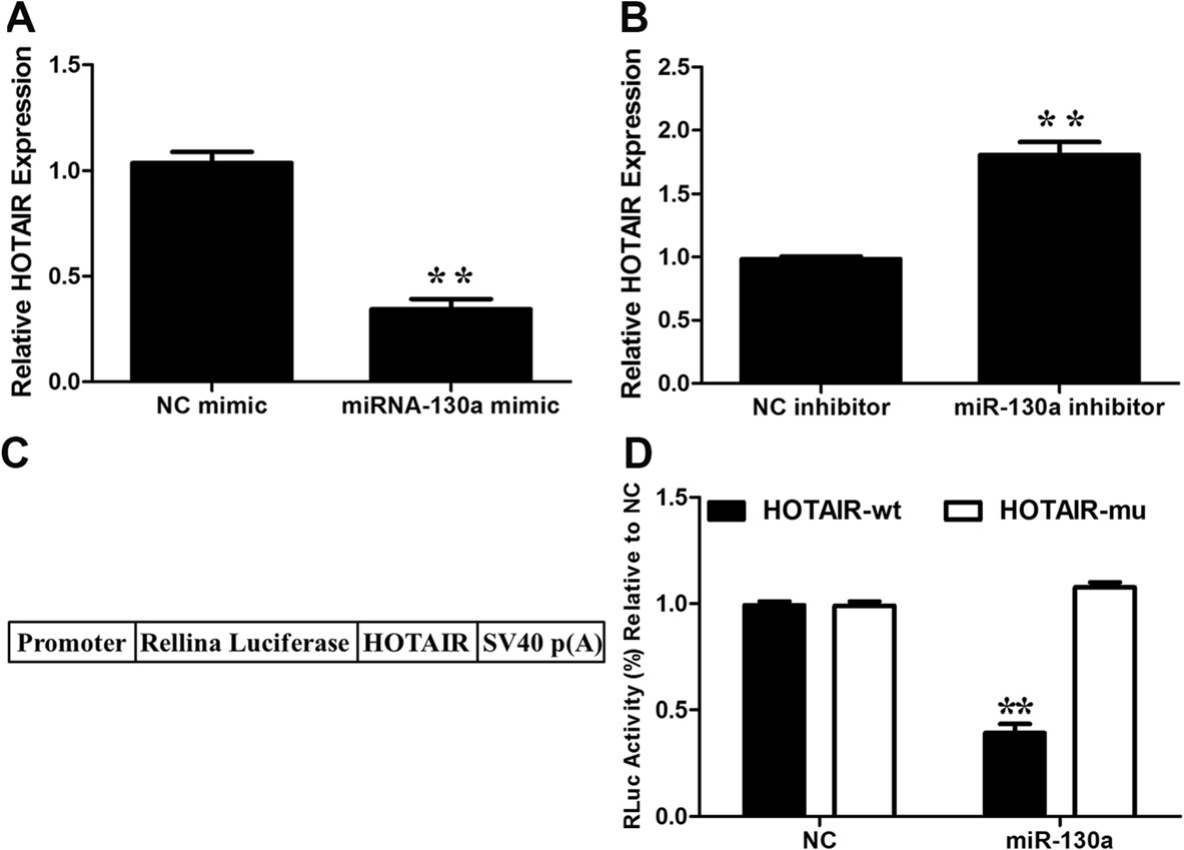
**Reciprocal negative regulation of miR-130a and HOTAIR. (A, B)** Effect of miR-130a on HOTAIR expression. GBC-SD cells were transfected with vector, miR-130a mimic **(A)** or miR-130a inhibitor **(B)**, and total RNA was isolated for qRT-PCR 24 h after transfection. **(C)** Schematic representation of the constructs generated for luciferase assays. **(D)** HOTAIR WT or its mutant devoid of specific miR-130a-binding sites in which seed matches were mutagenized from ‘TTGTAACGTGA’ to ‘GCGCCUCUUC’ was cloned downstream of Renilla luciferase gene (RLuc) in the vector pRL-TK and transfected into 293 T cells together with specific miRNAs mimics or the negative control mimic (NC). Luciferase assay was performed as described in Materials and Methods. Plotted are results from three independent experiments. Error bars represent S.E.M., n = 3. *p < 0.05; **p < 0.01, n.s., not significant.

The function of lncRNAs in human diseases may have to do with their ability to regulate gene expression. Recently, increasing evidence suggests that non-coding RANs may participate in ceRNA regulatory network [22]. For example, there is a negative correlation between loc285194 and miRNA-211 [16]. We performed a search for miRNAs with complementary base paring with HOTAIR utilizing online software program starbase v2.0 (http://starbase.sysu.edu.cn/mirLncRNA.php) [25]. We found that 20 miRNAs formed complementary base pairing with HOTAIR (Table 2). To determine whether any of them is truly regulated by HOTAIR as predicted, we profiled the expression of the 20 miRNAs in GBC-SD transfected with si-NC or si-HOTAIR. The initial profiling identified two miRNAs (miRNA-326, miRNA-130a) that were with a fold-change greater than 2 compared to the control (Table 3). We then focused on miRNA-130a (Figure 5A), which is of the greatest fold-change in response to HOTAIR knockdown.HOTAIR siRNAs significantly reduced the endogenous HOTAIR (Figure 5B); at the same time, HOTAIR siRNA increased the miRNA-130a level (Figure 5C, D). In contrast, ectopic expression of HOTAIR increased the expression level of HOTAIR (Figure 6A) while dramatically suppressed miRNA-130a (Figure 6B, C). To determine whether this suppression is through the potential interaction at the putative miRNA-130a-binding site, we generated a HOTAIR mutant (Figure 6D). This mutant HOTAIR clone revealed no significant suppression of miRNA-130a compared with wide-type of HOTAIR (Figure 6B, C). To determine whether miRNA-130a is able to negatively regulate HOTAIR, we also transfected miRNA-130a mimic into GBC-SD cells. As shown in Figure 7A, miRNA-130a mimic reduced the HOTAIR level by approximately 64%. Furthermore, miRNA-130a inhibitor increased the expression of HOTAIR (Figure 7B). To further confirm that the miR-130a target site is functional, luciferase reporter constructs were generated (Figure 7C). WT HOTAIR or HOTAIR mutant devoid of the miR-130a binding site was cloned downstream of Renilla luciferase gene and transfected into 293 T cells together with specific miR-130a mimics or the negative control mimic. The data revealed that luciferase expression was obviously reduced in cells transfected with HOTAIR and miR-130a mimics compared with that in cells transfected with HOTAIR and negative control. However, luciferase expression in cells transfected with HOTAIR mutant and the miR-130a mimics was comparable to that of control cells (Figure 7D). These data demonstrates that the binding sites are vital for the reciprocal repression of HOTAIR and miRNA-130a.

**Table 2:** miRNAs that have base-pairing with HOTAIR

	**miRNA**	**Target location**
1	hsa-miR-222-3p	chr12:54356181-54356201[-]
2	hsa-miR-206	chr12:54356155-54356176[-]
3	hsa-miR-221-3p	chr12:54356181-54356203[-]
4	hsa-miR-326	chr12:54360078-54360099[-]
5	hsa-miR-148a-3p	chr12:54356219-54356239[-]
6	hsa-miR-148b-3p	chr12:54356219-54356239[-]
7	hsa-miR-17-5p	chr12:54356298-54356320[-]
8	hsa-miR-20a-5p	chr12:54356298-54356320[-]
9	hsa-miR-130a-3p	chr12:54356628-54356649[-]
10	hsa-miR-19a-3p	chr12:54356629-54356655[-]
11	hsa-miR-330-5p	chr12:54360078-54360100[-]
12	hsa-miR-93-5p	chr12:54356298-54356322[-]
13	hsa-miR-106b-5p	chr12:54356298-54356319[-]
14	hsa-miR-106a-5p	chr12:54356298-54356320[-]
15	hsa-miR-761	chr12:54356646-54356666[-]
16	hsa-miR-214-3p	chr12:54356646-54356666[-]
17	hsa-miR-4295	chr12:54356628-54356645[-]
18	hsa-miR-613	chr12:54356155-54356174[-]
19	hsa-miR-19b-3p	chr12:54356629-54356652[-]
20	hsa-miR-152-3p	chr12:54356219-54356238[-]

**Table 3:** Initial profiling of miRNAs in response to knockdown of HOTAIR

	**miRNA**	**HOTAIR fold-change**
1	hsa-miR-222-3p	0.84
2	hsa-miR-206	0.60
3	hsa-miR-221-3p	0.77
4	hsa-miR-326	2.10
5	hsa-miR-148a-3p	1.64
6	hsa-miR-148b-3p	1.73
7	hsa-miR-17-5p	0.67
8	hsa-miR-20a-5p	0.92
9	hsa-miR-130a-3p	3.01
10	hsa-miR-19a-3p	0.56
11	hsa-miR-330-5p	1.35
12	hsa-miR-93-5p	0.71
13	hsa-miR-106b-5p	0.54
14	hsa-miR-106a-5p	0.59
15	hsa-miR-761	1.06
16	hsa-miR-214-3p	1.19
17	hsa-miR-4295	0.86
18	hsa-miR-613	1.01
19	hsa-miR-19b-3p	0.65
20	hsa-miR-152-3p	0.81

**Figure 8:**
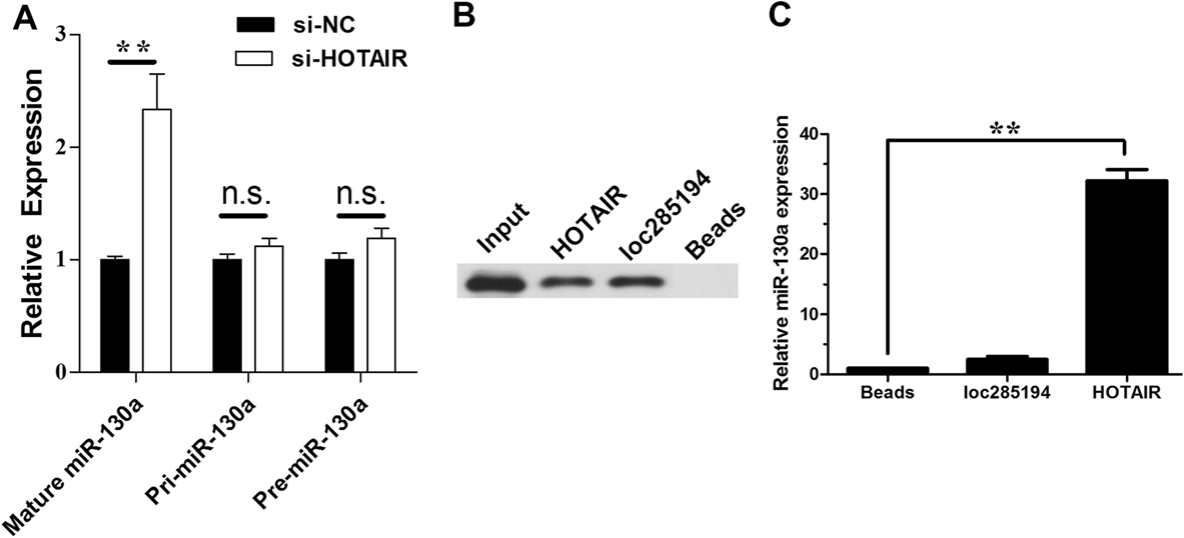
**The mechanism of the regulation of miR-130a by HOTAIR. (A)** Effect of HOTAIR on mature miR-130a, pri-miR-130a and pre-miR-130a. **(B)** Pull-down of Ago 2 by biotin-labeled HOTAIR or loc285194 RNA probe, as detected by western blot. The loc285194 lane was composed from the same gel with the same contrast. **(C)** Detection of miR-130a in the same pellet precipitated by the HOTAIR probe, but not in the pellet precipitated by the loc285194 probe. Error bars represent S.E.M., n = 3. *p < 0.05; **p < 0.01, n.s., not significant.

To explore the mechanism of the negative regulation of miRNA-130a by HOTAIR, we examined the effect of knockdown of HOTAIR on the expression level of mature miRNA-130a, pri-miRNA-130a and pre-miRNA-130a. As demonstrated in Figure 8A, while HOTAIR siRNA induced a significant upregulation of mature miRNA-130a, it had no effect on pri-miRNA-130a or pre-miRNA-130a, implying this negative regulation might be through a post-transcriptional mechanism. It is well known that miRNAs exert their gene silencing functions through a ribonucleoprotein complex called the RNA induced silencing complex (RISC) [26]. Potential microRNA targets can be isolated from this complex after Ago2 co-immunprecipitation [16,27] as Ago2 is a vital component of RISC complex necessary for siRNA or miRNA-mediated gene silencing. It does not exclude the possibility that miRNA-130a might be in separate RISC complexes even if we detected both miRNA-130a and HOTAIR in the Ago2 pellet with the Ago2 co-immunprecipitation assay. To determine whether miRNA-130a and HOTAIR are in the same RISC complex, we performed RNA pull-down experiments using HOTAIR probe and then examined Ago2 and miRNA-130a simultaneously as described previously [16,17]. A biotin-labeled HOTAIR RNA probe was synthesized and mixed with cellular extract [16]. We next performed in vitro RNA pull-down to validate the association between HOTAIR and Ago2. As a result, we detected Ago2 (Figure 8B). What’s more, we detected miRNA-130a in the same pellet (Figure 8C). Thus, these results indicate that both HOTAIR and miRNA-130a are probably in the same Ago2 complex.

### HOTAIR expression is positively correlated with c-Myc and negatively correlated with miRNA-130a in gallbladder cancer tissues

**Figure 9:**
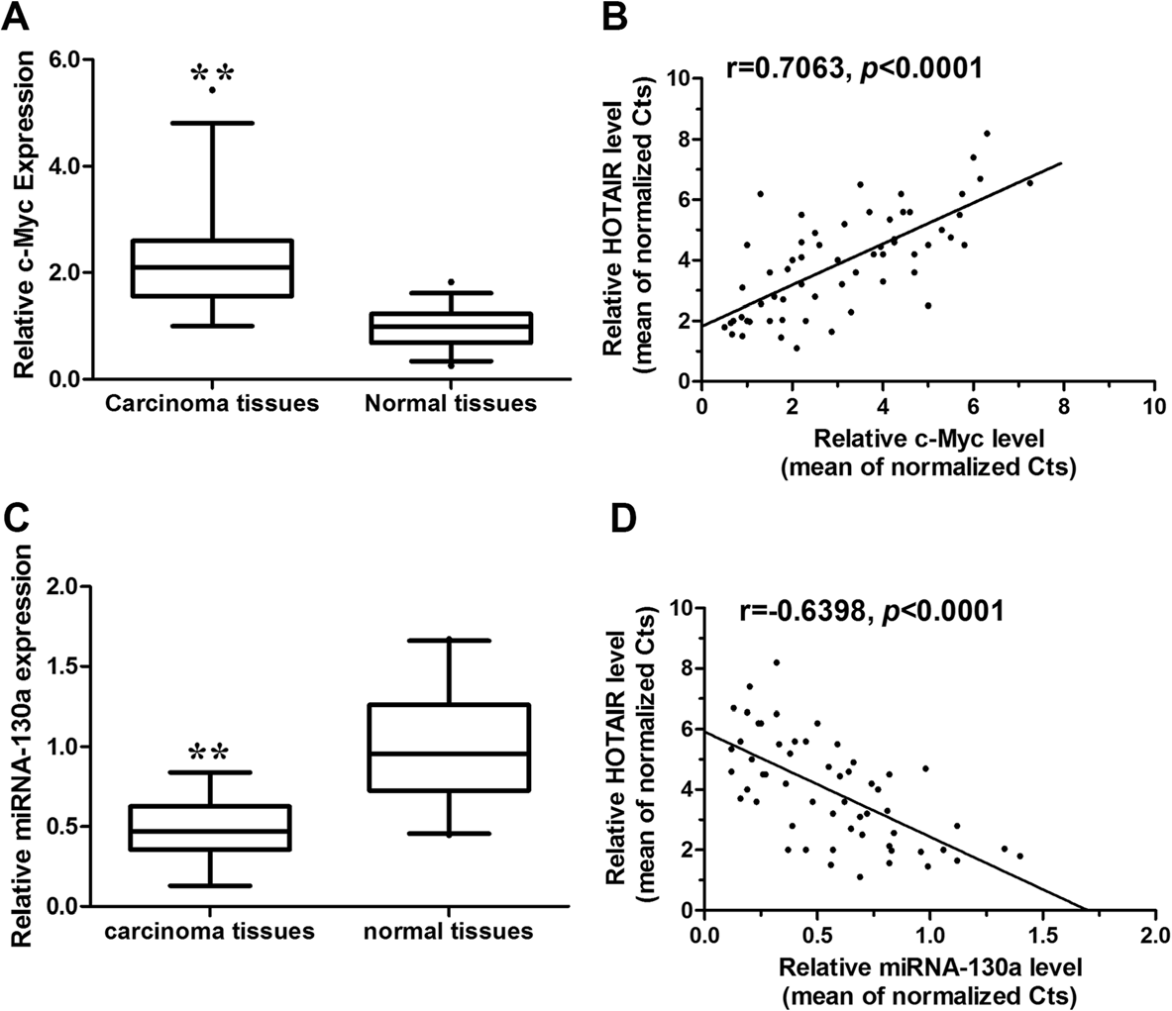
**Expression of HOTAIR, c-Myc and miRNA-130a mRNA levels in human gallbladder cancer samples. (A)** c-Myc is upregulated in gallbladder cancer tissues compared with paired adjacent normal gallbladder tissues. c-Myc mRNA expression was analyzed by real-time PCR and normalized to GADPH. The statistical differences between samples were analyzed with paired samples *t*-test (n = 65, *p* < 0.0001). **(B)** The correlation between HOTAIR and c-Myc expression levels in gallbladder cancer tissues and matched adjacent normal gallbladder tissues (n = 65). Quantitative RT-PCR was performed in triplicate for each sample and assays were repeated once. The relative levels were normalized to GADPH. Each point in the scatter graph represents an individual sample, in which relative c-Myc levels indicate on *x*-axis and HOTAIR levels on *y*-axis. The *x*-axis shows normalized Ct values for c-Myc determined by quantitative RT-PCR. The *y*-axis shows normalized Ct values for HOTAIR determined by quantitative RT-PCR. “Mean of normalized Ct values” is the subtraction of “mean of triple Ct values for c-Myc (*x*-axis) or HOTAIR (*y*-axis)” by “mean of triple Ct values for GADPH”. The correlation coefficient, R = 0.7063, *p* <0.0001, indicates there is a strongly positive relationship between c-Myc and HOTAIR. **(C)** miRNA-130a is downregulated in gallbladder cancer tissues compared with paired adjacent normal gallbladder tissues. miRNA-130a mRNA expression was analyzed by real-time PCR and normalized to GADPH. **(D)** The correlation between HOTAIR and miR-130a expression levels in gallbladder cancer tissues and matched adjacent normal gallbladder tissues (n = 65). The correlation coefficient, R = -0.6398, *p* <0.0001, indicates there is a strongly negative relationship between miRNA-130a and HOTAIR.

As c-Myc upregulates the expression of HOTAIR, we investigated whether a correlation exists between c-Myc and HOTAIR expression levels in gallbladder cancer tissues. We examined the expression level of c-Myc transcript in gallbladder cancer tissues from Figure 1. The c-Myc mRNA levels in cancer tissues were significantly higher than those in adjacent normal tissues (*p* < 0.0001, Figure 9A). A significant positive correlation was observed between HOTAIR and c-Myc mRNAs (*r* = 0.7063, *p* < 0.0001, Figure 9B), supporting the role of c-Myc in the expression of HOTAIR. We also determined the expression levels of miRNA-130a in gallbladder cancer tissues from Figure 1. The miRNA-130a mRNA was markedly downregulated in gallbladder cancer tissues compared to adjacent normal tissues (*p* < 0.0001, Figure 9C). HOTAIR expression was negatively correlated with miRNA-130a in gallbladder cancer tissues (*r* = -0.6398, *p* < 0.0001, Figure 9D), providing evidence to the reciprocal negative regulation of HOTAIR and miRNA-130a.

### HOTAIR’s oncogenic activity is in part through negative regulation of miRNA-130a

**Figure 10:**
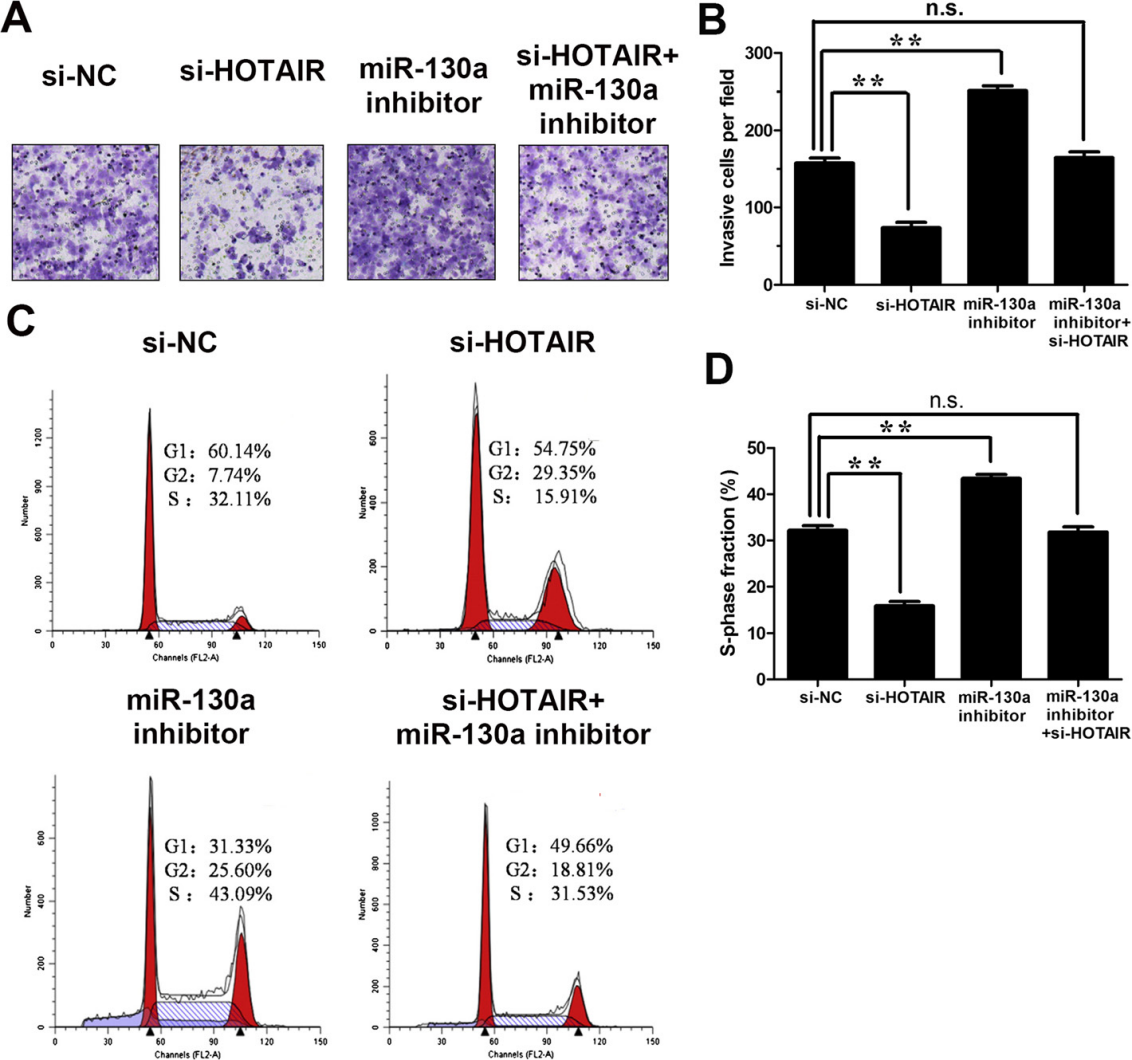
**HOTAIR’s oncogenic activity is in part through negative regulation of miRNA-130a.** Representative images **(A)** and the number of migratory cells **(B)** per high-power field transfected with si-NC, si-HOTAIR, miR-130a inhibitor or si-HOTAIR + miR-130a inhibitor. The migratory ability of GBC-SD cells can be blocked by HOTAIR downregulation. The si-HOTAIR-blocked migratory ability of GBC-SD cells was rescued by miR-130a inhibitor. Quantitative graphical representation of Apoptosis, G1, G2, S cell population **(C)** and a bar-graphical representation S-Phase Fraction cells in each group **(D)** transfected with si-NC, si-HOTAIR, miR-130a inhibitor or si-HOTAIR + miR-130a inhibitor. Si-HOTAIR induced a reduction of S-Phase Fraction cells in GBC-SD cells, which can be rescued by miR-130a inhibitor. Error bars represent S.E.M., n = 3. *p < 0.05; **p < 0.01.

To investigate the biological roles of HOTAIR and miRNA-130a in gallbladder cancer, we employed gain-of-function and loss-of-function approaches. We demonstrated that knockdown of HOTAIR inhibited the invasion of gallbladder cancer cells while miRNA-130a inhibitor reversed the decrease in invasiveness (Figure 10A, B). Flow cytometric analysis indicated knockdown of HOTAIR suppressed cancer cells proliferation (S-phase fraction) *in vitro* while miRNA-130a inhibitor rescued the proliferation (Figure 10C, D). These results may imply that the oncogenic activity of HOTAIR is partly through negative regulation of miRNA-130a.

## Discussion

As a new class of non-coding RNAs, long non-coding RNAs were found to be deregulated in a variety of diseases, especially cancer [13-15]. Understanding the precise molecular mechanism by which lncRNAs function is vital for exploring new potential strategies for early diagnosis and therapy. In this study, we present evidence that HOTAIR is a direct target of c-Myc and exhibits oncogenic activity partly through negative regulation of miRNA-130a. This study provides experimental evidence to the existence of the ceRNA regulatory network [22] where HOTAIR and miRNA-130a negatively regulate each other.

HOTAIR, a long non-coding RNA initially identified in breast cancer, was shown to be upregulated in a variety of carcinomas [15,28,29]. A large number of studies have focused on its biological role and association with clinical prognosis, yet the precise factors regulating its expression remains largely unknown expect that HOTAIR is transcriptionally regulated by estradiol in breast cancer [30], which is quite tumor specific. In this study, we predicted a putative binding site of c-Myc in the promoter region of HOTAIR. c-Myc is a well-known transcriptional factor, with its obligate heterodimerization partner Max, binds to DNA sequence elements called E-boxes to allow for target gene transcription [31]. Recent studies have suggested that c-Myc can regulate numerous protein-coding and non-coding genes, expecially miRNAs [32].

In this study, we demonstrated that c-Myc induced HOTAIR expression through direct interaction with the E-box in the HOTAIR promoter region. Ectopic expression of c-Myc increased HOTAIR expression and its promoter activity, while knockdown of c-Myc reduced HOTAIR expression and its promoter activity. Nucleotide mutant in the E-box element in the promoter region abrogated c-Myc-dependent promoter activation. The association of c-Myc with the HOTAIR promoter was confirmed by chromatin immunoprecipitation assays. What’s more, a positive correlation between c-Myc and HOTAIR mRNAs was observed in gallbladder cancer tissues, providing additional evidence to c-Myc’s regulation of HOTAIR. At present, two important lncRNAs have been shown to be c-Myc transcription targets [31,33], involved in the c-Myc mediated cellular process. Our study would strengthen the notion that, lncRNAs are also an important part of c-Myc regulatory network.

Previous studies suggested that HOTAIR interacts with PRC2 (polycomb repressive complex 2) and histone demethylases LSD1/CoREST/REST complexes through it 5’- and 3’-end, respectively. HOTAIR acts as a bridge coordinating the targeting of PRC2 and LSD1 complexes to chromatin for coupled histone H3K27 methylation and H3 lysine-4 (H3K4) demethylation processes, which leads to silencing of target genes [15,34]. A growing volume of recent work has established that lncRNAs can also regulate other non-coding RNAs, in particular miRNAs, and miRNAs may have an effect on the regulation of lncRNAs [18,19]. In support of this notion, we demonstrate that HOTAIR-mediated oncogenic activity is at least partly through suppression of miRNA-130a. Knockdown of HOTAIR induced the upregulation of miRNA-130a. Ectopic expression of HOTAIR reduced the miRNA-130a level and the miRNA-130a-binding site is vital for the HOTAIR-mediated repression. On the other hand, miRNA-130a inhibitor upregulated HOTAIR level while miRNA-130a mimic repressed HOTAIR level. HOTAIR and miRNA-130a may form a reciprocal repression feedback loop. In addition, a negative correlation was observed between HOTAIR and miRNA-130a in gallbladder cancer tissues, providing supporting evidence to such a feedback loop. In addition, we explored the mechanism of such a feedback loop. We found that HOTAIR and miRNA-130a bind to the same RISC complex. As miRNAs are known to mediate post-transcriptional control of gene expression by binding to the 3’-untranslated regions of protein coding genes, we suppose that the way that miR-130a promoted the downregulation of HOTAIR is somewhat similar to the miRNA-mediated silencing of protein-coding genes. HOTAIR is well-known for its induction of genome-wide targeting of the polycomb repressive complex 2 (PRC2), leading to an altered methylation of histone H3 lysine 27 (H3K27) and genes expression. Thus, we may hypothesize that HOTAIR may lead to the downregulation of miR-130a via increasing the methylation status of the promoter of miRNA-130a as Vrba et al. [35] demonstrated that the downregulation of miR-130a is linked to increased promoter methylation.

In this regard, HOTAIR may function as the endogenous sponge, similar to what has been reported for lncRNA loc285194 [16], GAS5 [17] and HULC [36]. For example, loc285184 is identified as a tumor-suppressive lncRNA in colon cancer. Loc285194 can downregulate miRNA-211 through its interaction with miRNA-211. Though much of the focus in ncRNA research is directed towards understanding the regulation of protein-coding genes mediated by them, it may seem that ncRNAs could form a well-orchestrated regulatory interaction network [18,22]. Our study further demonstrated that the reciprocial repression of HOTAIR and miRNA-130a is likely through the pathway involving RISC complex.

miRNA-130a has been found to be downregulated in a variety of carcinomas and exhibits tumor-suppressive activity [37-39] while HOTAIR was demonstrated be an oncogene and upregulated in carcinomas [28-30]. We then then studied that biological function of HOTAIR and miRNA-130a in gallbladder cancer cells. HOTAIR was shown to promote invasiveness and proliferation of cancer cells [28,29]. Our data showed that while knockdown of HOTAIR inhibited the invasiveness and proliferation of gallbladder cancer cells, miRNA-130a inhibitor reversed the effects that knockdown of HOTAIR exerted. These results provide additional evidence to the reciprocal repression loop in a functional aspect.

Our study suggests another layer of regulation that involves the ncRNAs. A better understanding of the ncRNA interaction regulatory network would deepen our understanding of the pathophysiological mechanism of various aspects of tumorigenesis including tumor growth, invasion, metastasis and chemo-resistance in gallbladder cancer.

## Competing interests

The authors declare that they have no competing interests.

## Authors’ contributions

MZM conceived of the study and participated in its design and coordinated and helped to draft the manuscript. MZM, CXL, YZ and MZW performed the experiments. MDZ and YYQ participated in the design of the study and performed the statistical analysis. MZM, ZWQ and WG wrote the paper. All authors read and approved the final manuscript.

## Authors’ information

Ming-zhe Ma, Chun-xiao Li and Yan Zhang are co-first authors

## Supplementary Material

Additional file 1 Primers used in this study. Click here for file

Additional file 2 The promoter of HOTAIR. Click here for file
