# Supplementary material for: Long non-coding RNA HOTAIR, a c-Myc activated driver of malignancy, negatively regulates miRNA-130a in gallbladder cancer
Source: Mol Cancer. 2014 Jun 23;13:156. doi: 10.1186/1476-4598-13-156 (PMC4085645; doi:10.1186/1476-4598-13-156)
Supplement: Additional file 1 — Primers used in this study. [file 1476-4598-13-156-S1.docx]

Primers used in this study

**qRT-PCR primers**

GADPH:

5’-GTCAACGGATTTGGTCTGTATT-3’ (forward), 5’-AGTCTTCTGGGTGGCAGTGAT-3’ (reverse);

c-Myc

5’-CACCAGCAGCGACTCTGA-3’ (forward),

5’-GATCCAGACTCTGACCTTTTGC-3’ (reverse);

HOTAIR:

5’-CAGTGGGGAACTCTGACTCG-3’ (forward), 5’-GTGCCTGGTGCTCTCTTACC-3’ (reverse)

miRNA-130a：

5’-GTCAGTGCAATGTTAAAAGGGCAT-3’ (forward), 5’-CAGTGCGTGTCGTGGAGT-3’ (reverse).

pri-miRNA-130a:

5’-GGTGGTCTCTGTGCTGGGGGTCAGG-3’ (forward), 5’-ATGCTGAGGAGGCAGCCAGCGCTGGGTAG-3’ (reverse).

pre-miRNA-130a:

5’-TGCTGCTGGCCAGAGCTC-3’ (forward),

5’-CACTACACGGCCAATGCCC-3’ (reverse).

**To clone HOTAIR or c-Myc**

HOTAIR

sense:5’-CATGGATCCACATTCTGCCCTGATTTCCGGAACC-3’,

antisense: 5’-ACTCTCGAGCCACCACACACACACAACCTACAC-3’.

c-Myc

sense: 5’-CGAATTCCCGCGACGATGCCCCT-3’,

antisense: 5’-GCTCTAGATTTCCTTACGCACAAGAGTTCCG-3’

**siRNA for HOTAIR or c-Myc**

HOTAIR

target sequence: siRNA，5’-AAAUCCAGAACCCUCUGACAUUUGC-3’

c-Myc siRNA

target sequence: 5’-GGUGAUCCAGACUCUGACCUU-3’

**To clone HOTAIR promoter**

5’-ACTAAGCTTATCTTTCTTTGCTAGCTGC-3’ (forward),

5’-ACTTCTAGATTTCCAGCGTTCTCTGGGCG-3’ (reverse)

**For ChIP assay**

HOTAIR promoter containing E-box

5’-GCGGAGAGAGTCCCACACA-3’ (forward),

5’-GGGATTATTGCAGGCAGTG-3’ (reverse)

non-E-box

5’-GGTGGGATTGCCCTGTTGG -3’ (forward),

5’-CCGCGAGCTCACTTTGATC-3’ (reverse)

**For luciferase reporter assay**

a point mutation in E-box

5’-ACTGGTACCTAAGCGGAGAGAGTCCCACACAGG-3’ (forward) 5’-ACTAAGCTTGAGTCAGAGTTCCCCAC-3’ (reverse)
