# Supplementary figures and images for: Long non-coding RNA HOTAIR, a c-Myc activated driver of malignancy, negatively regulates miRNA-130a in gallbladder cancer
Source: Mol Cancer. 2014 Jun 23;13:156. doi: 10.1186/1476-4598-13-156 (PMC4085645; doi:10.1186/1476-4598-13-156)

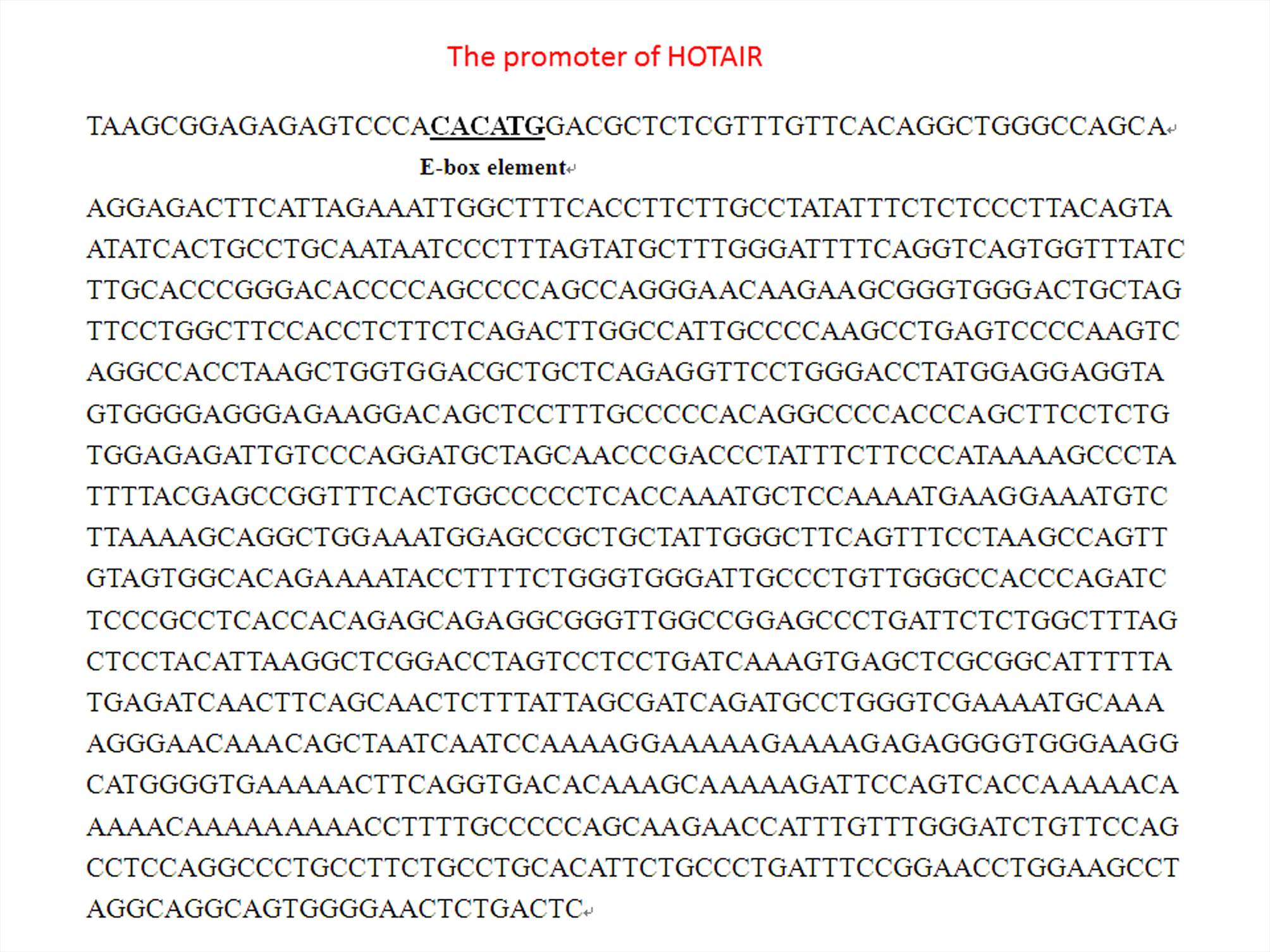

Supplement: Additional file 2 — The promoter of HOTAIR. [file 1476-4598-13-156-S2.tiff]
